# MicroRNA miR-125a-3p modulates molecular pathway of motility and migration in prostate cancer cells

**DOI:** 10.18632/oncoscience.30

**Published:** 2014-04-30

**Authors:** Lihi Ninio-Many, Hadas Grossman, Mattan Levi, Sofia Zilber, Ilan Tsarfaty, Noam Shomron, Anna Tuvar, Dana Chuderland, Salomon M Stemmer, Irit Ben-Aharon, Ruth Shalgi

**Affiliations:** ^1^ Department of Cell and Developmental Biology, Sackler Faculty of Medicine, Tel-Aviv University, Israel; ^2^ Institute of Oncology, Davidoff Center, Rabin Medical Center, Beilinson Campus, Petah-Tiqva, and Sackler School of Medicine, Tel Aviv University, Israel; ^3^ Department of Pathology, Rabin Medical Center, Beilinson Campus, Petah-Tiqva, Israel; ^4^ Department of Clinical Microbiology and Immunology, Sackler School of Medicine, Tel Aviv University, Israel.; ^5^ This work was performed in partial fulfillment of the requirements for a Ph.D. degree of Lihi Ninio-Many, Sackler Faculty of Medicine, Tel Aviv University, Israel.

**Keywords:** miR-125a-3p, Fyn, migration, prostate cancer, EMT, live imaging, actin cytoskeleton

## Abstract

Fyn kinase is implicated in prostate cancer. We illustrate the role of miR-125a-3p in cellular pathways accounted for motility and migration of prostate cancer cells, probably through its regulation on Fyn expression and Fyn-downstream proteins. Prostate cancer PC3 cells were transiently transfected with empty miR-Vec (control) or with miR-125a-3p. Overexpression of miR-125a-3p reduced migration of PC3 cells and increased apoptosis. Live cell confocal imaging indicated that overexpression of miR-125a-3p reduced the cells' track speed and length and impaired phenotype. Fyn, FAK and paxillin, displayed reduced activity following miR-125a-3p overexpression. Accordingly, actin rearrangement and cells' protrusion formation were impaired. An inverse correlation between miR-125a-3p and Gleason score was observed in human prostate cancer tissues. Our study demonstrated that miR-125a-3p may regulate migration of prostate cancer cells.

## INTRODUCTION

Prostate cancer is the most common cancer in men and a leading cause of male death from cancer in Western countries. Though current treatments for prostate cancer are effective, a considerable percent of the treated population may relapse, displaying a castrate-resistant phenotype, and thus rendering the essential need for new avenues of therapies.

Src family kinases (SFKs), members of the Tyrosine Kinasess family, are pleiotropic activators, proto-oncogenes. They play crucial roles in regulation of cell proliferation and cytoskeleton rearrangement [1] by mediating extracellular interactions driven by various molecules, as G protein-coupled receptor (GPCR), c-Met, EGFR, androgen receptor (AR) and integrins [2-4]. SFKs exhibit marked tumorigenic potential in the progression of prostate cancer, both paracrine-induced and cell autonomous-initiated, thus placing them as unique potential targets for anti-cancer therapy [5, 6].

Fyn, a member of the SFKs, is known as an important mediator of mitogenic signals and a regulator of cells' proliferation, adhesion and motility [7-9]. Fyn is up-regulated in prostate cancer where it exhibits tumorigenic potential in processes of cellular motility. Its interaction with several signal molecules, including focal adhesion kinase (FAK), Akt and paxillin, that play an essential role in prostate cancer progression, might account for the described cell transformations and possibly lends credence to its role in both cancer progression and metastasis [10-12].

MicroRNAs (miRNAs) are evolutionally conserved, small, endogenously expressed RNAs, which can silence gene expression by binding the 3′ untranslated regions (UTRs) of target mRNAs. They act by repressing translation or directing mRNA degradation [13]. A large number of miRNAs were reported to be involved in cancer progression showing an aberrant and variable level of expression [14, 15]. Furthermore, several studies demonstrated that the expression patterns of miRNAs can reflect the trait of the disease, its response to treatment and the risk for its progression [16, 17]. miRNA-125a (miR-125a) was demonstrated to regulate major cellular processes in several types of cancer cells. Over-expression of miR-125a impaired migration and invasion of breast cancer cells [18]. In hepatocellular carcinoma cells, ectopic expression of miR-125a resulted in inhibition of proliferation and metastases, caused by miR-125a's ability to target matrix metalloproteinase 11 (MMP11) and vascular endothelial growth factor A [19]. A role of miR-125a in the process of invasion and migration has also been implied in gastric and lung cancer cells [20, 21].

In a former study performed on human embryonic kidney (HEK 293T) cell-line, we have established the role of miR-125a-3p, an isoform derived from the 3′ arm of pre-miR-125a, in regulating cell migration and proliferation accomplished by targeting Fyn [22]. Moreover, we reported that miR-125a-3p reduced the activity of Fyn-downstream proteins as FAK, paxillin and Akt. In light of the above, along with studies that established the involvement of Fyn in processes of cellular motility in prostate cancer [10, 11], we demonstrate in the current study that miR-125a-3p down-regulates cellular pathways that account for proliferation and migration of prostate cancer cells, and portray its regulation of key proteins involved in these pathways. We also show an inverse correlation between the expression of miR-125a-3p and the Gleason score in tissue samples obtained from patients diagnosed with prostate cancer. Our results imply that miR-125a-3p modulates molecular pathways of motility and migration in prostate cancer cells.

## RESULTS

### miR-125a-3p impairs cell cycle, viability and induces apoptosis

**Figure 1:**
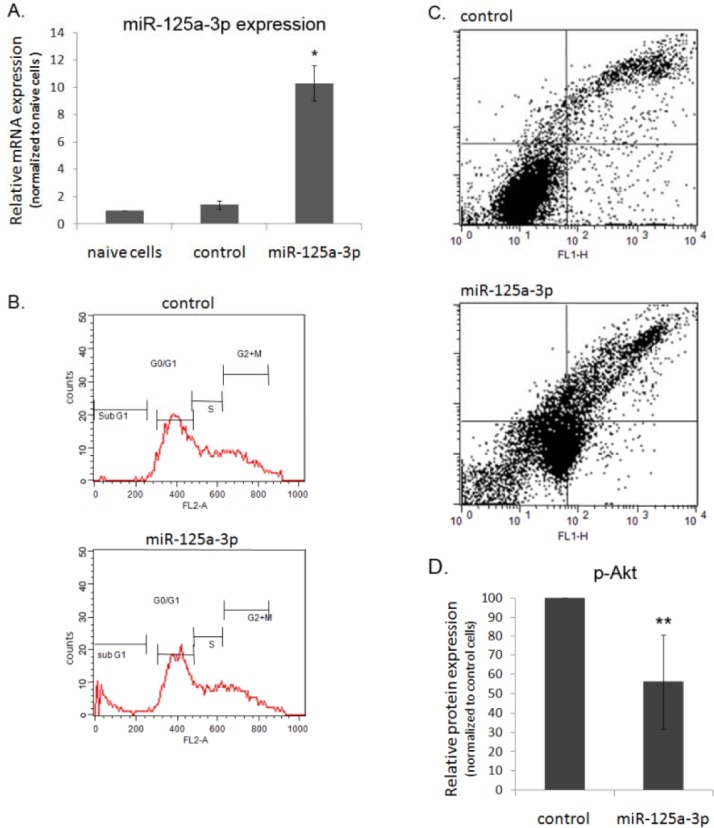
miR-125a-3p impairs cell cycle, viability and induces apoptosis (A) Endogenous and manipulated expression of miR-125a-3p. The expression level of miR-125a-3p was measured in PC3 naive cells, in cells transfected with miR-125a-3p or with empty vector (control), by Real-Time PCR using primers against miR-125a-3p and U6-SnRNA as a calibrator. (B-D) PC3 cells were transfected with miR-125a-3p or with a control vector, collected 48 hours later and subjected to: (B) Flow cytometry for DNA content. Representative FACS diagrams of one of three independent experiments. (C) Flow cytometry for annexin-V and PI labeling; percent of cells labeled with annexin-V (early apoptosis, bottom right square) or with a combination of annexin-V and PI (late apoptosis, upper right square), was measured. (D) WB analysis of phospho-Akt (p-Akt) and its loading control, general Akt. The experiment was repeated 3 times. Intensity of bands was analyzed using the image J software and the ratio between p-Akt and general Akt is presented. The bars are mean+SD. (*; P<0.05 and **; p<0.01) - Significantly different from control value.

PC3 human prostate cancer cell line, derived from an androgen-refractory prostate cancer, was used as a model in the current study. Initially we demonstrated that miR-125a-3p is endogenously expressed in PC3 cells. Ectopic expression of miR-125a resulted in overexpression of miR-125a-3p. Transfection with a control plasmid (an empty miR-Vec that contained no miRNA; see Methods) had no effect on the level of miR-125a-3p and was therefore used as a transfection control in the following experiments (Figure 1A).

In order to evaluate the effect of miR-125a-3p on cells' viability, we preformed FACS analysis, which indicated that overexpression of miR-125a-3p led to a 10 fold increase in the percentage of cells at the sub G1 stage whereas other stages of the cell cycle were not affected (Figure 1B). It also indicated, when using annexin-V and PI staining, a 4 fold increase and a 1.4 fold increase in the percentage of cells undergoing early and late apoptosis (Figure 1C; bottom right square and top right square, respectively). Reinforced by western blot (WB) analysis that showed reduced activity of Akt-1 protein, an anti-apoptotic factor, in cells overexpressing miR-125a-3p (Figure 1D), our results imply that miR-125a-3p induces apoptosis.

### miR-125a-3p alters migration and morphology of PC3 cells

**Figure 2:**
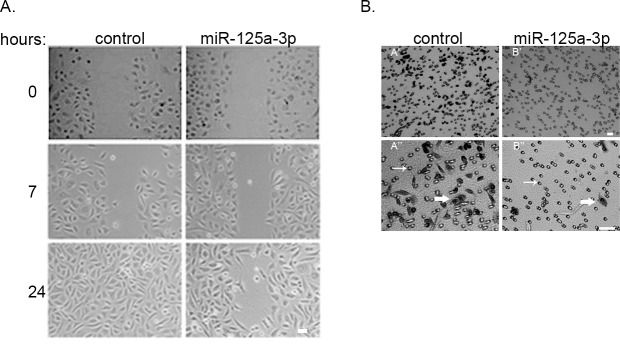
miR-125a-3p impairs cells motility Representative pictures of PC3 cells transfected with miR-125a-3p or empty miR-Vec (control), and subjected, 48 hours later, to cell migration assays: (A) scratch assay (B) transwell assay. Thick arrows indicate the migrating cells. A' and B'- lower magnification photos of control and miR-125a-3p overexpressing cells; A'' and B''- higher magnification photos, respectively. Thin arrows point at the membrane pores. Bar =50μm. The results were analyzed using the image J software. Similar results were obtained in three independent experiments.

We initially employed a scratch assay to evaluate whether miR-125a-3p affects migration. A significant decrease in the migratory capacity of the cells was observed 24 hours after performing the scratch; miR-125a-3p overexpressing cells covered only 48% of the scratch area whereas control cells migrated and covered the entire scratch area (Figure 2A). However, since a scratch assay does not take into account the apoptotic cells, we performed a transwell assay in which it is possible to exclude the apoptotic cells by seeding a similar number of viable control or miR-125a-3p-overexpressing cells. We found a 34% decrease in the number of miR-125a-3p-overexpressing cells that migrated for 6-8 hours through the pores towards the serum underneath the plate, compared to control cells (Figure 2B).

**Figure 3:**
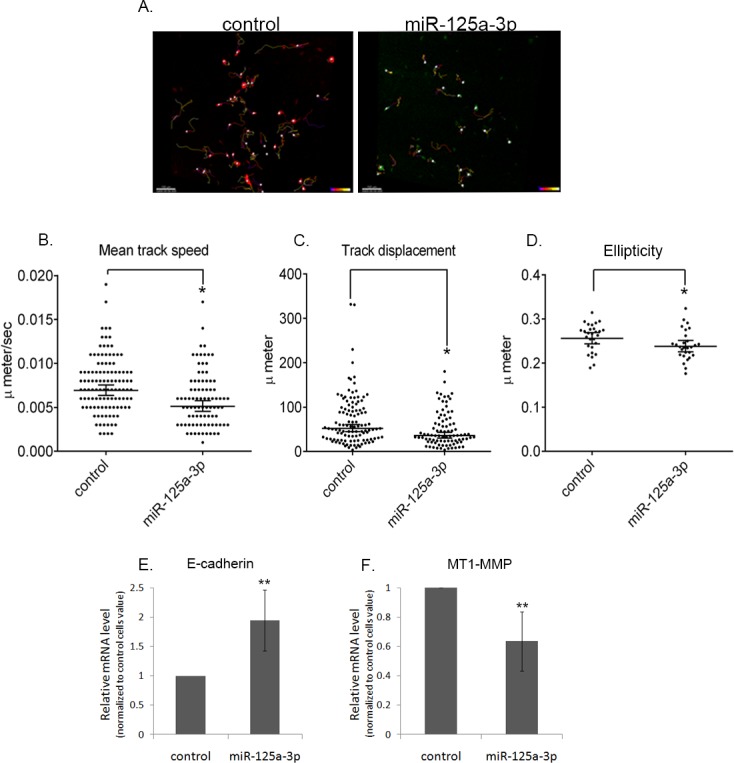
miR-125a-3p impairs the morpho-kinetic coordinated collective migration PC3 cells were co-transfected with either: miR-125a-3p and GFP plasmid or an empty miR-Vec and RFP plasmid (control). Twenty four hour after the transfection, the two cell populations were mixed, cultured for an additional 24 hours and then imaged by live confocal microscopy. Image stacks were taken every 5 minutes during 15 hours. The images were processed and analyzed by the Imaris software. (A) Representative pictures of imaged miR-125a-3p overexpressing cells (miR-125a-3p; right) and control cells (left). Each white dot represents one cell. Each colored line indicates the track of one cell. Graphs summarize (B) Track lengh, (C) Track speed and (D) Elipticity of 111 control cells and 86 miR-125a-3p-overexpressing cells. All parameters collected along the experiments were analyzed for each cell and only then, group analysis (control or miR-125a-3p) was performed. Results were analyzed by one-sample T-test; p<0.05. PC3 cells, transfected for 48 hours with miR-125a-3p or with empty miR-Vec (control), were subjected to qPCR analysis with specific primers for (E) E-cadherin or (F) MT1-MMP; all calibrated with the endogenous control, HPRT1. The experiment was repeated 3 times and analyzed by one-sample T-test. Bars are mean+SD. (*) - Significantly different from control cells value (p<0.05).

To further delve into the effect of miR-125a-3p on migration, we performed quantitative measurements of cell behavior and followed the trajectory and shape of individual cells by live cell confocal imaging. To this end, cells were co-transfected with control vector and an RFP plasmid (Figure 3A, left), or with miR-125a-3p and a GFP plasmid (Figure 3A, right; supplementary I). Remarkably, cells overexpressing miR-125a-3p presented a 28% decrease in their mean track speed (Figure 3B) and their track displacement was ~30% shorter than that of control cells (Figure 3C). Moreover, we also observed a phenotypic change in the shape of the cells: control cells presented a more elongated shape, resembling epithelial to mesenchymal transition (EMT) phenotype, whereas cells overexpressing miR-125a-3p acquired a round shape, resembling epithelial phenotype (Figure 3D). The latter phenotype was reinforced by real-time polymerase chain reaction (qPCR) analysis showing an almost two fold increase (94%) in the level of E-cadherin mRNA (an epithelial marker; Figure 3E), whereas the level of N-cadherin mRNA remained unchanged (not shown); and a 37% decrease in the level of MT1-MMP mRNA (Figure 3F). These results suggest that miR-125a-3p plays a key role in modulating cells migration by a mechanism that involves regulation of EMT.

### miR-125a-3p suppresses the level of expression of Fyn mRNA and protein, as well as of its downstream effectors

**Figure 4:**
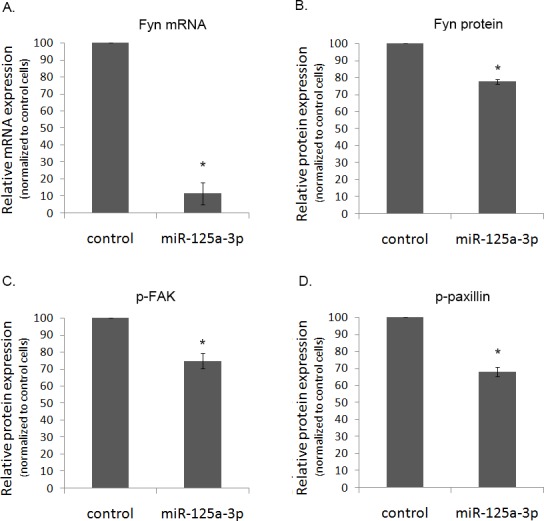
The effect of miR-125a-3p on hallmark genes in prostate cancer PC3 cells, transfected with miR-125a-3p or with empty vector (control), were cultured for 48 hours. Cells were lysed and, (A) their Fyn mRNA expression was analyzed by Real Time PCR. Proteins were analyzed by WB with specific antibodies against: (B) Fyn, (C) phospho-FAK (p-FAK) and (D) phospho-paxillin (p-paxillin), as well as against their loading control proteins (actin, general FAK and general paxillin, respectively). The experiment was repeated 3 times. Intensity of bands was analyzed using the image J software and the ratio between each protein and its control was plotted. The bars are mean+SD. (*) - Significantly different from control value (p <0.05).

To further understand the molecular mechanism by which miR-125a-3p inhibits migration, we examined its effect on several key proteins. We showed that miR-125a-3p reduced the expression level of Fyn mRNA and protein (Figure 4A and B), respectively. We then examined the effect on the activity of Fyn's downstream effectors, FAK and paxillin, both of which have a key role in cell spreading and motility. WB analysis indicated a 26% and 32% decrease in the phosphorylation state of FAK and paxillin in miR-125a-3p-overexpressing cells (Figure 4C and D), respectively.

### miR-125a-3p impairs the actin cytoskeleton

**Figure 5:**
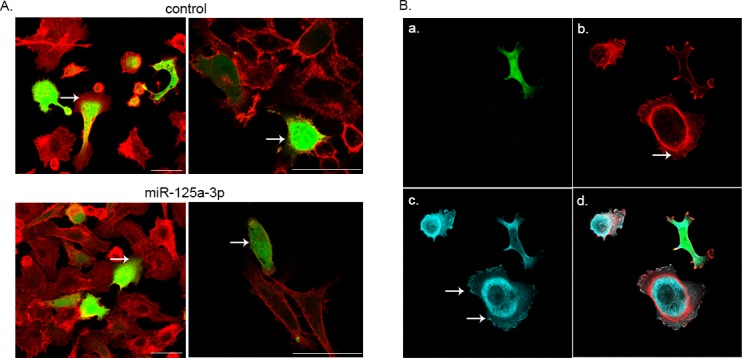
Polymerization of actin filaments PC3 cells were seeded onto coverslips and (A) co-transfected with miR-125a-3p and GFP (bottom panel; green) or with empty miR-Vec and GFP (control; top panel; green). Cells were fixed 48 hours later and processed for F-actin staining using FITC-conjugated phalloidin (red). The arrows point at the actin cytoskeleton in the cell. Bar = 50μm. (B) co-transfected with miR-125a-3p and GFP (green; a.), fixed 48 hours later and stained with anti-paxillin antibody (blue; c; arrows point at focal adhesion sites) and anti F-actin FITC-conjugated phalloidin (red;b; arrow points at actin filaments). Merge (d.).

The fact that miR-125a-3p reduced the cells' migratory capability and the activity of FAK and paxillin, two proteins that serve as anchors for actin filaments [23], prompted us to examine whether the inhibition of cell migration by miR-125a-3p was mediated by alterations in actin cytoskeleton. We therefore transfected PC3 cells with either miR-125a-3p and GFP or with control vector and GFP (as a control), and then stained for actin filaments (F-actin) using FITC-conjugated phalloidin. With the aid of a confocal microscope we observed an impairment of the actin rearrangement in cells overexpressing miR-125a-3p, along with a decrease in their membrane protrusions which are necessary for cell migration (Figure 5A). We further showed that overexpression of miR-125a-3p hampers the focal adhesion sites. Cells co-transfected with miR-125a-3p and GFP presented an impaired anchorage of the actin filament tips to the focal adhesion sites that showed no staining of paxillin, compared to control cells (Figure 5B).

Taken together, our results suggest that miR-125a-3p reduces cell membrane protrusions probably by impairing the dynamic interplay between the actin cytoskeleton and cell adhesion sites, leading to reduction in cell motility.

### miR-125a-3p is reduced in prostate cancer samples

**Figure 6:**
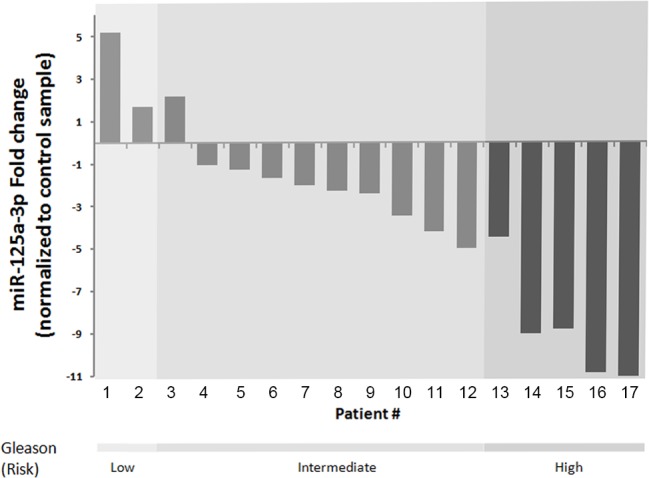
miR-125a-3p expression is reduced in human prostate cancer The expression of miR-125a-3p was studied in Formalin-Fixed, Paraffin-Embedded (FFPE) human prostate cancer samples. Prostate tissues of patients with histologically confirmed prostate cancer stored at the Department of Pathology at the Rabin Medical Center were included in the study. Only tissue samples containing more than 50% tumor and larger than 0.5 cm in diameter that enable analysis of both tumor and its adjacent normal tissue were used in this study. miRNAs were extracted and purified from FFPE tissues by the FFPE extraction kit (Qiagen). The results, of 17 patients, are presented as the fold change of the expression of miR-125a-3p in tumor sample compared to its normal control (non pathologic sample). Low risk - Gleason 6, intermediate risk - Gleason 7, high risk- Gleason 8-10. Each column represents one patient. p<0.05 using Wilcoxon test, P value is statistically significant for difference between high and intermediate and low Gleason score.

The expression pattern of miR-125a-3p was analyzed in biopsies of prostate tissue samples from 20 patients diagnosed with prostate cancer. Comparing the cancerous tissue with adjacent non-cancerous tissue revealed an inverse correlation between miR-125a-3p expression and Gleason score of the tissue; we dichotomously defined the samples according to the pathological known score of “risk of recurrence” and could delineate an inverse correlation between tumor differentiation and miR-125a expression (Figure 6A; p<0.05 Wilcoxon test). Patient's characteristics are presented in table1 Sup.

## DISCUSSION

In this study we elucidate the role of miR-125a-3p in cellular pathways accounted for motility and migration of prostate cancer cells. Following our analysis of non-cancerous HEK 293T cells at which the regulation of Fyn expression by miR-125a-3p has been established [22], we confirmed that this regulation applies also in prostate cancer cell.In view of former evidence of miRNA's critical role in tumor invasion and metastasis [24] we focused on studying the potential involvement of miR-125a-3p in cellular pathways leading to motility and migration of cancer cells. We demonstrated that over-expression of miR-125a-3p inhibited the migratory capability of PC3 cells.

The process of cell migration and invasion may occur in two modes: one, a mesenchymal motility mode that involves dynamic changes in the rearrangement of actin cytoskeleton and its interplay with focal adhesion proteins that induces membrane protrusions mandatory for movement. The other, an amoeboid motility mode that facilitates detachment of cells from the primary tumor [25]. The migratory ability of cancer cells is dependent upon their ability to undergo EMT [26]. EMT correlates specifically with metastasis and increased invasiveness of several cell lines [27], and is associated with a shift in the expression levels of known proteins such as cadherins [28] (a decrease in E-cadherin and an increase in N-cadherin). We show that over expression of miR-125a-3p led to an increase in E-cadherin expression, as well as a decrease in the expression of MT1-MMP, a matrix metalloproteinase known to induce EMT in prostate cancer cells [29]. Furthermore, we also noticed that the morphology of miR-125a-3p-overexpressing cells differed from that of control cells; the miR-125a-3p-overexpressing cells acquired a round shape that resembled epithelial cells rather than mesenchymal cells. Our results imply that high levels of miR-125a-3p might interfere with EMT in prostate cancer cells.

To further analyze the morpho-kinetic parameters of migration in miR-125a-3p overexpressing cells, we employed confocal live imaging. When comparing miR-125a-3p-overexpressing cells to controls, we were able to show a significant reduction in known migration parameters as track displacement and mean track speed, suggesting that overexpression of miR-125a-3p inhibited the cells' motility.

Cell motility involves a complex series of events that promote remodeling of actin cytoskeleton, mediated by various stimuli and translated into establishing actin rich protrusions required for movement [30]. Herein we confirm an impairment of F-actin organization resulting in disarrangement of actin-derived protrusions in cells overexpressing miR-125a-3p; a phenomenon that may underlie the impairment of the migratory capability as observed in our study. The ability of miR-125a-3p to hamper the actin cytoskeleton and thereby to inhibit cells migration has also been shown in lung cancer cells [21]. However, it was suggested that this effect of miR-125a-3p is mediated by miR-125a-3p-induced decrease in the expression of RhoA, a well-known regulator of actin cytoskeletal and adhesion dynamics [31]. Based on the established interaction between miR-125a-3p, Fyn [22] and on former studies characterizing the correlation between Fyn and the cytoskeleton [32, 33], we propose that miR-125a-3p may alter the dynamics of actin cytoskeleton arrangement via a Fyn-dependent pathway. Taken together, our results show that miR-125a-3p impairs the rearrangement of the actin filaments, the activity of FAK and paxillin [23, 34], and the localization of paxillin to the focal adhesions sites at the tips of the actin filaments. This implies that miR-125a-3p reduces the cell membrane protrusions probably by impairing the dynamic interplay between the actin cytoskeleton and cell adhesion sites, thus leading to reduced cell motility.

miR-125a-3p may also regulate cell migration by an ancillary pathway - the PI3K/Akt signaling pathway. Activated Akt is known to regulate downstream signaling proteins involved in cell survival, cell growth and cell cycle progression. Overexpression of phosphorylated Akt accompanied advanced human prostate cancer [35]. A recent study demonstrated its role in cells' migration, showing that ablation of Akt1 inhibited PC3 cell migration [36]. Though the mechanism by which Akt exerts this activity is not well understood; its expression led to up-regulation of Fyn and concomitantly prompted the invasion of H-Ras-enhanced-human keratinocyte cells and K-Ras mutated-human breast cancer cells [7, 37]. The interaction between Fyn and Akt was shown to be reciprocal; Fyn induced Akt activation in an indirect manner [37] and hampered the activation of Akt when it was down-regulated in HEK 293T cells [22] We demonstrate that overexpression of miR-125a-3p led to a reduction in the activity of Akt, which may have an impact on both proliferation and migration via signaling pathways that involve Fyn.

We find the interpretation that miR-125a-3p hampers cells migratory capability by inducing apoptosis, as a plausible one, though we assume that this effect is not a direct result of apoptosis because the effect of miR-125a-3p on cells migration was greater than its effect on apoptosis. This assumption is based also on our results indicating that miR-125a-3p-overexpressing viable cells present reduced migration in transwell assay as well as down-regulation of migration-correlated migratory proteins. We, therefore suggest that miR-125a-3p affects both proliferation and migration via partially-linked pathways.

Since bioinformatics does not predict FAK, paxillin or Akt as miR-125a-3p targets, we can conclude that miR-125a-3p may exert its effect on these proteins via Fyn. However, since a single miRNA may encompass multiple targets, alternative pathways should be further explored.

Former evidence indicate that cell migration is regulated by SFKs in general and by Fyn in particular [7, 38, 39]. Moreover, Fyn is located downstream of the HGF/Met signaling pathway and impacts cellular tropism and shape in prostate cancer cell line [11]. Recently it has been demonstrated that aggressive breast cancer cells overexpressing high levels of endogenous Met exhibited constitutive, Met-dependent membrane blebbing, through Rho-ROCK pathway, and induced cell motility as well; both phenomena are utilized by cancer cells for migration and metastasis [40]. Our results imply a role for miR-125a-3p in regulating cell migration, possibly, in part, via a Fyn dependent manner. Other targets of miR-125a-3p as chemokine (C-C motif) ligand 4 and IGF-2, may also be involved in this process, as has already been suggested [41].

Finally, we sought for clinical implication for our findings, and therefore analyzed the expression pattern of miR-125a-3p in prostate tissue samples retrieved from patients diagnosed with prostate cancer and compared them with the adjacent non-cancerous tissue. We observed an inverse correlation between miR-125a-3p and Gleason score.

Our results are compatible with former studies that indicated down-regulation of miR-125a-3p in human gastric and lung cancer [41, 42]. We demonstrate herein that miR-125a-3p remarkably impaired the migratory capability of PC3 cells, as seen by dynamic confocal imaging of miR-125a-3p transfected PC3 cells. This result corresponds highly with the inverse correlation of miR-125a-3p expression and Gleason score in human prostate cancer tissues. Since high Gleason score correlates with higher risk for disease recurrence and metastatic potential caused by primary tumor cells spread, miR-125a-3p that plays a key role in modulating migration pathways accounting for the high metastatic potential, may be of high clinical relevance. Thus, miR-125a-3p may represent a new plausible therapeutic target to reduce metastatic potential of prostate cancer and to define high risk patients for preventive therapy. Future studies should address the cellular mechanism underlying miR-125a-3p involvement in modulating migratory capacity of prostate cancer cells and its potential role in an *in vivo* model.

## METHODS

### Primary antibodies

anti Fyn (sc-16), and anti phospho-paxillin (sc-101774; Santa Cruz Biotechnology, Santa Cruz, CA, USA). Anti - and anti general-Akt (#9721 and #2938, respectively; Cell signaling Technology, Danvers, MA, USA). Anti FAK (#AHO0502; Biosource International (Camarillo, CA, USA). Anti phospho-FAK (#44625G; Invitrogen, Carlsbad, CA, USA). Anti actin (#MAB1501; Millipore, Temecula, CA, USA). Anti paxillin (#610052; BD Transduction Laboratories, San Diego, CA, USA). Rodhamine Phalloidin (Molecular Probes, Eugene, OR,USA)

### Secondary antibodies

monoclonal and polyclonal HRP-conjugated antibodies (Jackson Immunoresearch, West Grove, PA, USA). Goat anti mouse, 647 – labeled (#28175; Anaspec, San Jose, CA, USA).

### Cell culture and transfection

Adherent cultures of PC3 cell line were maintained in RPMI medium (Biological Industries, Beit-Ha'emek, Israel) supplemented with 10% FCS (Biological Industries) and antibiotics. The cells were incubated in a humidified atmosphere of 5% CO_2_ in air at 37 °C. Cells were seeded onto 6-well plates (35 mm; Nunc, Copenhagen, Denmark) at a density of 8×10^5^ cells/well and transfected 24 hours later.

Transfection was performed using Lipofectamin 2000 Transfection Reagent according to manufacturer's instructions (Invitrogen). Complete medium was added 24 hours after transfection, for an additional 24 hours, before subjecting the cells to subsequent analysis.

### Immunoblotting (WB)

Cells were lysed for 20 minutes in ice-cold radio-immuno-precipitation assay buffer (RIPA; 20mM TrisHCl pH 7.4, 137mMNaCl, 10% glycerol, 1% Triton X-100, 0.5% sodium deoxycholate, 0.1% SDS, 2mM EDTA pH 8, 2 mM vanadate, 1 mM PMSF and a cocktail of protease inhibitors; Boehringer, Mannheim, Germany). Cells' lysate was cleared by centrifugation and an appropriate sample buffer was added. Samples were subjected to sodium dodecyl sulfate polyacrylamide gel electrophoresis (SDS-PAGE), immunoblotted with the appropriate primary antibodies (anti Fyn 1:300, anti phospho-FAK 1:1000, anti FAK 1:100, anti phospho-paxillin 1:1000, anti paxillin 1:1000, anti phospho-Akt 1:1000, Anti Akt 1:1000 or anti actin 1:10,000), incubated with the corresponding horseradish peroxidase-conjugated secondary antibodies and subjected to enhanced chemiluminescence assay (ECL; Thermo Scientific, Rockford, IL, USA). The intensity of the bands was analyzed by the Image J software.

### RNA isolation, reverse transcription (RT) and real-time polymerase chain reaction (qPCR)

Total RNA was extracted by Trizol (Invitrogen) according to manufacturer's instructions. Reverse transcription (RT) for gene expression or miRNA expression was carried out by high capacity cDNA RT kit (Applied Biosystems, Foster City, CA, USA; 10- 50ng RNA fractions). All RT reactions were carried out by a StepOnePlus Real-Time PCR System (Applied Biosystems).

For gene expression - the reactions were conducted using SYBR Green dye (Applied Biosystems) according to the manufacturer's insrtuctions. The following primers were used for the analysis:

Fyn (forward primer: 5′-GGACATGGCAGCACAGGTG-3′, reverse primer: 5′-TTTGCTGATCGCAGATCTCTATG-3′),

MT1-MMP (forward primer: 5′-GCC ACC AGG AAG ATG TCA TT -3′, reverse primer: 5′-GAT GCA CAG TGG CAC CTT C -3′),

E-cadherin (forward primer: 5′-TTG ACG CCG AGA GCT ACA C -3′, reverse primer: 5′-GTC GAC CGG TGC AAT CTT -3′),

N-cadherin (forward primer: 5′-CTC CAT GTG CCG GAT AGC-3′, reverse primer: 5′- CGA TTT CAC CAG AAG CCT CTA C)

Hypoxanthine phosphoribosyltransferase 1 (HPRT1) as endogenous control (forward primer: 5′-TGACACTGGCAAAACAATGCA-3′, reverse primer: 5′-GGTCCTTTTCACCAGCAAGCT-3′).

For miRNA expression - miR-125a-3p (Assay ID: 2199) and U6-snRNA (AssayID: 001973) were measured by the TaqMan miRNA kit (Applied Biosystems) according to the manufacturer's instructions. Mature miRNAs were normalized to U6-snRNA. Relative expression was calculated using the comparative Ct.

### Immunofluorescence staining

PC3 cells were cultured on 13-mm round glass coverslips (Marienfeld GmbH, Germany). After the desired treatment, culture medium was aspirated, cells were washed three times with cold PBS, fixed for 30 minutes in 3% paraformaldehyde and permeabilized for additional 30 minutes by a permeabilization solution (0.1% TritonX-100, 5% FCS and 2% bovine serum albumin [BSA; Sigma, Chemical Company, St. Louis, MO, USA] in PBS). Cells were incubated for 1 hour at room temperature with rodhamine-phalloidin for actin labeling (Molecular Probes; 1:150), washed and mounted with Gel Mount (Sigma) or incubated with anti paxillin antibody (BD Transduction Laboratories, 1:100), washed, incubated with secondary antibody (Anaspec, 1:400) and folllowed by incubation with rodhamine-phalloidin, washed and mounted. Cells samples were analyzed using an LSM 510, Zeiss laser confocal scanning microscope (Carl Zeiss, Oberkochen, Germany) or with (Stimulated Emission Depletion) Leica TCS STED microscope (Leica, Wetzlar, Germany).

### Cell cycle analysis

Following the desired treatments, cells were subjected to trypsin, washed 3 times in cold phosphate buffered saline (PBS), re-suspended in 1.0 ml hypotonic buffer (50 μg/ml propidium iodide, 0.1% sodium citrate and 0.1% Triton X-100) and incubated for at least 1 hour at 4°C in the dark. The DNA content of the cells was measured by a fluorescence-activated sorter (Becton Dickinson FACSort Flow Cytometer, San Jose, CA, USA) and analyzed using the WinMDI 2.8 software.

### MTT cell proliferation assay

Transfected cells were seeded at 2x104cells/well onto 24-well plates to a final volume of 100μl and incubated for 48 hours. Ten μl of MTT (3-(4, 5-Dimethylthiazol-2-yl)-2, 5-diphenyltetrazolium bromide; 5mg/ml) were added to each well before an additional 2-4 hours incubation period at 37°C. The reaction was terminated by 110μl HCl (0.07M in isopropanol) and OD 560_nm_ was measured by the SpectraMax 190 microplate reader (Molecular Probes).

Migration assays -Transwell assay - PC3 cells (overexpressing miR-125a-3p or empty miR-Vec) were stained with trypan blue and counted in a Countess automated cell counter machine (Invitrogen). Live cells (2*10^5^) were pre-incubated in FCS-free DMEM at the upper wells of Transwell plate (24 wells, 8 μm pore size membranes, Corning 3422; Corning, NY, USA) for migration assay. After 6 hours, 350μl of DMEM with 20% FCS as a chemo-attractant were added to the lower well and cells were allowed to migrate during a 24 hours period of incubation at 37C; 5% CO2 in air. Cells, attached to both sides of the membrane, were washed twice with PBS. Cells on the upper side of the membrane were removed by cotton swabs, and the migrating cells at the bottom of the membrane were visualized by a fluorescence microscope, photographed and counted by the Image J software. Scratch assay - Cells were allowed to grow to confluent monolayers; when approaching 100% cell confluence, the monolayers were wounded by scratching the surface, as uniformly as possible, with a pipette tip. The initial wounding and the movement of the cells over the scratched area were monitored at different time points using the Nikon ECLIPSE Ti microscope (Nikon Instruments, Melville, NY, USA).

### Live cell imaging

PC3 cells were seeded in 6-well plate and then co-transfected with miR-125a-3p together with a GFP plasmid or with an empty miR-Vec together with an RFP plasmid (control cells). The transfected cells were trypsinized 24 hours later and mixed together in a glass-bottom plate for additional 24 hours. For live cell imaging, the plate was maintained within a microscope chamber on a heated microscope stage at 37°C, 5% CO_2_ and the cells were imaged continuously for 15 hours by the Zeiss-510 confocal laser-scanning microscope (Carl Zeiss). The image stacks were processed and parameters of cell's migration and shape were analyzed by Imaris software using the autoregressive motion algorithm (Bitplane, South Windsor, CT, USA). (Max gap size = 3, max distance between measurements = 100 μm, only continuous tracks, longer than 2.5 hours, were included in the analysis).

### Extraction of miRNA from Formalin-Fixed, Paraffin-Embedded (FFPE) human prostate cancer samples

Formalin-Fixed, Paraffin-Embedded prostate tissues of patients with histologically confirmed prostate cancer, stored at the Department of Pathology at Rabin Medical Center (RMC) were included in the study. Tissue sections were accompanied by clinical data retrieved from the database of Davidoff Center at the RMC. The protocol was approved by the Institutional Review Board at RMC (No. 6083). We have implemented the method for purification of miRNAs from FFPE sections of human prostate cancer samples. miRNAs were extracted from the cancerous prostate tissue and from adjacent non-cancerous tissue using miRNeasy FFPE kit (Qiagene, GmbH, Hilden, Germany) according to the manufacturer's instructions and the expression of miR-125a-3p was determined by qPCR as described earlier.

### Data analysis and statistics

Data is expressed as mean+SD. Individual comparisons were made using a one-sample T-test. Tissue samples were analyzed by Wilcoxon test. P value of 0.05 was considered statistically significant.

## References

[1] Parsons SJ, Parsons JT (2004). Src family kinases, key regulators of signal transduction. Oncogene.

[2] Thomas SM, Brugge JS (1997). Cellular functions regulated by Src family kinases. Annu Rev Cell Dev Biol.

[3] Tang X, Feng Y, Ye K (2007). Src-family tyrosine kinase fyn phosphorylates phosphatidylinositol 3-kinase enhancer-activating Akt, preventing its apoptotic cleavage and promoting cell survival. Cell Death Differ.

[4] Prag S, Lepekhin EA, Kolkova K, Hartmann-Petersen R, Kawa A, Walmod PS, Belman V, Gallagher HC, Berezin V, Bock E, Pedersen N (2002). NCAM regulates cell motility. J Cell Sci.

[5] Cai H, Smith DA, Memarzadeh S, Lowell CA, Cooper JA, Witte ON (2011). Differential transformation capacity of Src family kinases during the initiation of prostate cancer. Proc Natl Acad Sci U S A.

[6] Kim LC, Song L, Haura EB (2009). Src kinases as therapeutic targets for cancer. Nat Rev Clin Oncol.

[7] Yadav V, Denning MF (2011). Fyn is induced by Ras/PI3K/Akt signaling and is required for enhanced invasion/migration. Mol Carcinog.

[8] Kim HJ, Warren JT, Kim SY, Chappel JC, DeSelm CJ, Ross FP, Zou W, Teitelbaum SL (2010). Fyn promotes proliferation, differentiation, survival and function of osteoclast lineage cells. J Cell Biochem.

[9] Lewin B, Siu A, Baker C, Dang D, Schnitt R, Eisapooran P, Ramos DM (2010). Expression of Fyn kinase modulates EMT in oral cancer cells. Anticancer Res.

[10] Posadas EM, Al-Ahmadie H, Robinson VL, Jagadeeswaran R, Otto K, Kasza KE, Tretiakov M, Siddiqui J, Pienta KJ, Stadler WM, Rinker-Schaeffer C, Salgia R (2009). FYN is overexpressed in human prostate cancer. BJU Int.

[11] Jensen AR, David SY, Liao C, Dai J, Keller ET, Al-Ahmadie H, Dakin-Hache K, Usatyuk P, Sievert MF, Paner GP, Yala S, Cervantes GM, Natarajan V, Salgia R, Posadas EM (2011). Fyn is downstream of the HGF/MET signaling axis and affects cellular shape and tropism in PC3 cells. Clin Cancer Res.

[12] Saito YD, Jensen AR, Salgia R, Posadas EM (2010). Fyn: a novel molecular target in cancer. Cancer.

[13] Bartel DP (2004). MicroRNAs: genomics, biogenesis, mechanism, and function. Cell.

[14] Volinia S, Calin GA, Liu CG, Ambs S, Cimmino A, Petrocca F, Visone R, Iorio M, Roldo C, Ferracin M, Prueitt RL, Yanaihara N, Lanza G, Scarpa A, Vecchione A, Negrini M (2006). A microRNA expression signature of human solid tumors defines cancer gene targets. Proc Natl Acad Sci U S A.

[15] Xi JJ (2013). MicroRNAs in Cancer. Cancer Treat Res.

[16] Shen J, Stass SA, Jiang F (2013). MicroRNAs as potential biomarkers in human solid tumors. Cancer Lett.

[17] Cho WC (2010). MicroRNAs: potential biomarkers for cancer diagnosis, prognosis and targets for therapy. Int J Biochem Cell Biol.

[18] Scott GK, Goga A, Bhaumik D, Berger CE, Sullivan CS, Benz CC (2007). Coordinate suppression of ERBB2 and ERBB3 by enforced expression of micro-RNA miR-125a or miR-125b. J Biol Chem.

[19] Bi Q, Tang S, Xia L, Du R, Fan R, Gao L, Jin J, Liang S, Chen Z, Xu G, Nie Y, Wu K, Liu J, Shi Y, Ding J, Fan D (2012). Ectopic expression of MiR-125a inhibits the proliferation and metastasis of hepatocellular carcinoma by targeting MMP11 and VEGF. PLoS One.

[20] Nishida N, Mimori K, Fabbri M, Yokobori T, Sudo T, Tanaka F, Shibata K, Ishii H, Doki Y, Mori M (2011). MicroRNA-125a-5p is an independent prognostic factor in gastric cancer and inhibits the proliferation of human gastric cancer cells in combination with trastuzumab. Clin Cancer Res.

[21] Huang B, Luo W, Sun L, Zhang Q, Jiang L, Chang J, Qiu X, Wang E (2013). MiRNA-125a-3p is a negative regulator of the RhoA-actomyosin pathway in A549 cells. Int J Oncol.

[22] Ninio-Many L, Grossman H, Shomron N, Chuderland D, Shalgi R (2013). microRNA-125a-3p reduces cell proliferation and migration by targeting Fyn. J Cell Sci.

[23] Turner CE (2000). Paxillin interactions. J Cell Sci.

[24] Dalmay T, Edwards DR (2006). MicroRNAs and the hallmarks of cancer. Oncogene.

[25] Berton S, Belletti B, Wolf K, Canzonieri V, Lovat F, Vecchione A, Colombatti A, Friedl P, Baldassarre G (2009). The tumor suppressor functions of p27(kip1) include control of the mesenchymal/amoeboid transition. Mol Cell Biol.

[26] Thiery JP (2002). Epithelial-mesenchymal transitions in tumour progression. Nat Rev Cancer.

[27] Gotzmann J, Mikula M, Eger A, Schulte-Hermann R, Foisner R, Beug H, Mikulits W (2004). Molecular aspects of epithelial cell plasticity: implications for local tumor invasion and metastasis. Mutat Res.

[28] Wheelock MJ, Shintani Y, Maeda M, Fukumoto Y, Johnson KR (2008). Cadherin switching. J Cell Sci.

[29] Cao J, Chiarelli C, Richman O, Zarrabi K, Kozarekar P, Zucker S (2008). Membrane type 1 matrix metalloproteinase induces epithelial-to-mesenchymal transition in prostate cancer. J Biol Chem.

[30] Ridley AJ (2011). Life at the leading edge. Cell.

[31] Merajver SD, Usmani SZ (2005). Multifaceted role of Rho proteins in angiogenesis. J Mammary Gland Biol Neoplasia.

[32] Levi M, Kaplan-Kraicer R, Shalgi R (2011). Regulation of division in mammalian oocytes: implications for polar body formation. Mol Hum Reprod.

[33] Talmor-Cohen A, Tomashov-Matar R, Tsai WB, Kinsey WH, Shalgi R (2004). Fyn kinase-tubulin interaction during meiosis of rat eggs. Reproduction.

[34] Chen M, Chen SC, Pallen CJ (2006). Integrin-induced tyrosine phosphorylation of protein-tyrosine phosphatase-alpha is required for cytoskeletal reorganization and cell migration. J Biol Chem.

[35] Malik SN, Brattain M, Ghosh PM, Troyer DA, Prihoda T, Bedolla R, Kreisberg JI (2002). Immunohistochemical demonstration of phospho-Akt in high Gleason grade prostate cancer. Clin Cancer Res.

[36] Cariaga-Martinez AE, Lopez-Ruiz P, Nombela-Blanco MP, Motino O, Gonzalez-Corpas A, Rodriguez-Ubreva J, Lobo MV, Cortes MA, Colas B (2013). Distinct and specific roles of AKT1 and AKT2 in androgen-sensitive and androgen-independent prostate cancer cells. Cell Signal.

[37] Gilhooly EM, Rose DP (1999). The association between a mutated ras gene and cyclooxygenase-2 expression in human breast cancer cell lines. Int J Oncol.

[38] Roche S (1998). [Tyrosine kinases of the Src family, enzymes with multiple functions: from the growth of fibroblasts to the migration of epithelial cells]. C R Seances Soc Biol Fil.

[39] Cary LA, Chang JF, Guan JL (1996). Stimulation of cell migration by overexpression of focal adhesion kinase and its association with Src and Fyn. J Cell Sci.

[40] Laser-Azogui A, Diamant-Levi T, Israeli S, Roytman Y, Tsarfaty I (2013). Met-induced membrane blebbing leads to amoeboid cell motility and invasion. Oncogene.

[41] Jiang L, Huang Q, Zhang S, Zhang Q, Chang J, Qiu X, Wang E (2010). Hsa-miR-125a-3p and hsa-miR-125a-5p are downregulated in non-small cell lung cancer and have inverse effects on invasion and migration of lung cancer cells. BMC Cancer.

[42] Hashiguchi Y, Nishida N, Mimori K, Sudo T, Tanaka F, Shibata K, Ishii H, Mochizuki H, Hase K, Doki Y, Mori M (2012). Down-regulation of miR-125a-3p in human gastric cancer and its clinicopathological significance. Int J Oncol.

